# Effectiveness of a Multicomponent Formulation: A Prospective Observational Study in Patients with Gastroesophageal Reflux Symptoms

**DOI:** 10.3390/ph19060866

**Published:** 2026-05-30

**Authors:** Giulia Fiorini, Luigi Gatta, Matteo Pavoni, Gabriella Massarenti, Beatrice Rosa, Cristina Marchesani, Giulia Collatuzzo, Raffaele Manta, Luciano Potena, Attilio Varricchio, Arrigo Francesco Giuseppe Cicero, Federica Fogacci, Claudio Borghi, Giovanni Barbara, Dino Vaira

**Affiliations:** 1 Cardiovascular Medicine Unit, IRCCS Azienda Ospedaliero-Universitaria di Bologna, 40138 Bologna, Italy; giulia.fiorini@aosp.bo.it (G.F.); luciano.potena2@unibo.it (L.P.); arrigo.cicero@unibo.it (A.F.G.C.); claudio.borghi@unibo.it (C.B.); 2 Department of Medical and Surgical Sciences, University of Bologna, 40126 Bologna, Italy; matteo.pavoni2@unibo.it (M.P.); gabriella.massarenti@studio.unibo.it (G.M.); beatrice.rosa6@studio.unibo.it (B.R.); cristina.marchesani@studio.unibo.it (C.M.); federica.fogacci@studio.unibo.it (F.F.); giovanni.barbara@unibo.it (G.B.); 3 Management Staff Department Area of Organizational Innovation Complex Operational Unit for Outpatient and Diagnostic Demand Management, Tuscany North West Local Health Authority, 56121 Pisa, Italy; gattalg@gmail.com; 4 Department of Biomedical and Clinical Sciences, University of Milan, 20157 Milan, Italy; giulia.collatuzzo@unimi.it; 5 Gastroenterology and Digestive Endoscopy, “Riuniti” Hospital, Azienda USL Toscana Nord Ovest, 57124 Livorno, Italy; raffaelemanta4@gmail.com; 6 Department of Otolaryngology, University of Molise, 86100 Campobasso, Italy; attilio.varricchio@unimol.it

**Keywords:** gastroesophageal reflux disease (GERD), Tamacid-Pro^®^, dyspepsia

## Abstract

**Background:** Gastroesophageal reflux symptoms (GERSs) represent the most prevalent phenotype of gastroesophageal reflux disease and frequently overlap with the symptoms of functional dyspepsia, posing diagnostic and therapeutic challenges. Limitations of long-term acid suppression have prompted interest in alternative mucosa-protective approaches. This study was conducted to evaluate the effectiveness of a novel multicomponent formulation (Tamacid-Pro^®^) in patients with reflux-like symptoms and negative endoscopy. **Methods:** In this prospective observational study, consecutive adult patients undergoing upper gastrointestinal endoscopy at a tertiary centre between January 2025 and February 2026 were screened. Patients with upper gastrointestinal symptoms lasting ≥2 months, negative endoscopy, and no evidence of *Helicobacter pylori* infection were enrolled. Participants received Tamacid-Pro^®^ three times daily for 3 months. Symptom frequency and intensity were assessed at baseline and after treatment using the Reflux Disease Questionnaire (RDQ). Changes over time were analyzed using paired *t*-tests, and multivariable linear regression was performed to identify response predictors. **Results:** A total of 1035 patients were included. After 3 months of treatment, all RDQ items showed a statistically significant improvement in both frequency and intensity (*p* < 0.0001). Significant reductions were observed in the GERD composite score, as well as in heartburn, regurgitation, and dyspepsia dimensions (all *p* < 0.0001). In a multivariable analysis, female sex was independently associated with greater improvement across multiple symptom domains, whereas alcohol consumption was negatively associated with improvement in the heartburn dimension. **Conclusions:** In this large real-world cohort of endoscopy-negative patients, treatment with Tamacid-Pro^®^ was associated with significant improvement in both typical reflux and dyspeptic symptoms. These findings support the role of multicomponent, mucosa-protective formulations as a valuable therapeutic option in patients with GERSs and overlapping functional gastrointestinal disorders.

## 1. Introduction

Gastroesophageal reflux disease (GERD) and dyspepsia are highly prevalent disorders in the general population, with pooled prevalence estimates of about 15% and 21%, respectively [1,2,3,4,5]. Beyond their frequency, both disorders are associated with significant impairment in quality of life, reduced work productivity, and substantial healthcare utilization [6]. The major clinical manifestations of GERD are heartburn and regurgitation. The mechanisms underlying gastroesophageal reflux symptoms are complex and likely multifactorial, involving disturbances such as reduced lower esophageal sphincter pressure, transient sphincter relaxations, hiatal hernia, impaired gastric emptying, and heightened visceral sensitivity [1,2,3,4].

Dyspepsia, by contrast, refers to symptoms thought to arise from the gastroduodenal region, in keeping with the Rome diagnostic framework. In a minority of patients, dyspeptic symptoms may be explained by organic conditions such as peptic ulcer disease or, less commonly, upper gastrointestinal malignancy [7]. In most cases, however, no structural abnormality is identified, and the condition is classified as functional dyspepsia. The pathophysiology of functional dyspepsia is heterogeneous and remains incompletely understood [8]. Proposed mechanisms include visceral hypersensitivity and delayed gastric emptying, both of which are also implicated in reflux-related symptom generation. Additional factors that may contribute include impaired gastric accommodation, altered central processing of pain, prior infectious gastroenteritis, and chronic *Helicobacter pylori* (*H. pylori*) infection [7]. An overlap between gastroesophageal reflux symptoms and dyspepsia has been reported, suggesting that these disorders frequently coexist and are associated with impaired quality of life [9]. Attempting to reliably differentiate these two entities by relying solely on medical history or standardized symptom questionnaires is notoriously difficult and frequently proves impossible in a routine setting [1,3]. A definitive diagnosis requires a negative upper endoscopy combined with ambulatory 24 h esophageal pH monitoring, but this approach is not routinely used outside of specialized centres [1,3]. As a result, an empiric trial of proton-pump inhibitors (PPIs) is commonly adopted in clinical practice [1,10]. Management is usually guided by symptom severity and frequency. Lifestyle and dietary measures remain first-line interventions [4,11,12]. When these behavioural shifts prove insufficient, PPIs become the established, evidence-based cornerstone of therapy for those with gastroesophageal reflux symptoms (GERSs) and EPS [2,4,11,12,13]. However, long-term acid suppression has recognized limitations, including drug interactions, restricted use in selected populations, and economic costs [14]. Advances in the understanding of GERD pathophysiology, with particular regard to impaired mucosal integrity, have driven the development of novel topical esophageal therapies that act by mitigating reflux and by forming a stable, resilient protective film over the esophageal mucosa [15].

In this context, Tamacid-Pro^®^ (Levante, Sesto Fiorentino, Italy) is a multicomponent dietary supplement developed to manage reflux symptoms. It was introduced into the Italian market in 2023, and it has since been increasingly adopted in clinical settings, given the growing interest in mucosa-protective approaches to managing gastroesophageal reflux symptoms. The formulation contains Gingigel Pro^®^ (Petit Medical Group, via della Pescara 16, Teramo, Italy), sodium alginate, *Tamarindus indica*, and plant extracts. Gingigel Pro^®^ is a patented matrix composed of xanthan gum, ginger extracts enriched in 6-gingerol and 6-shogaol, sodium and potassium citrates, and naringin. The formulation is designed to support gastrointestinal motility and improve palatability by reducing the pungency of ginger. These components may contribute to symptom control through complementary mechanisms, including antacid activity, modulation of mucosal sensitivity, enhancement of gastric emptying, raft formation, and mucosal protection [16,17,18,19,20]. Notably, this study provides the first clinical evidence on the use of this novel formulation.

The aim of this study was, therefore, to evaluate the clinical effectiveness of Tamacid-Pro^®^ in endoscopy-negative patients with heartburn, regurgitation, and/or epigastric pain or burning.

## 2. Results

**Figure 1:**
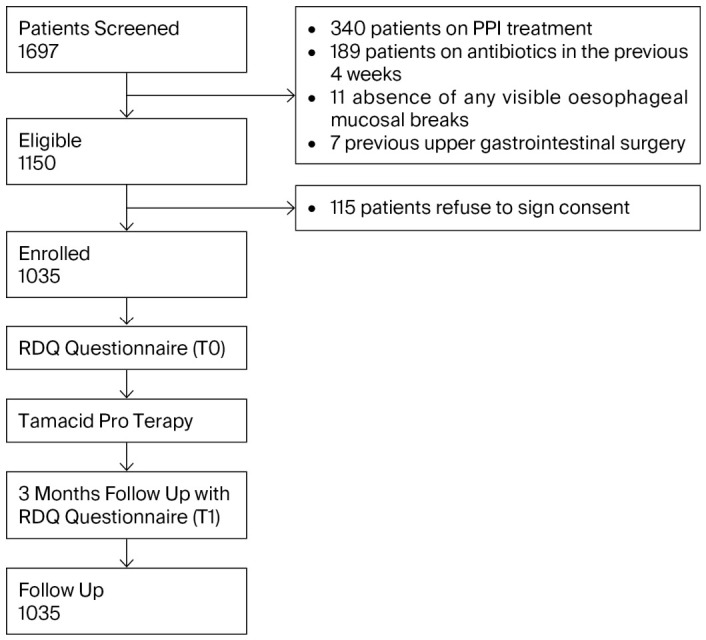
Flowchart of the study.

Between January 2025 and February 2026, 1697 patients were assessed for eligibility. Of these, 1150 were considered potentially eligible, and 1035 consented to participate in the study (Figure 1). The characteristics of the 1035 enrolled patients are shown in Table 1.

**Figure 2:**
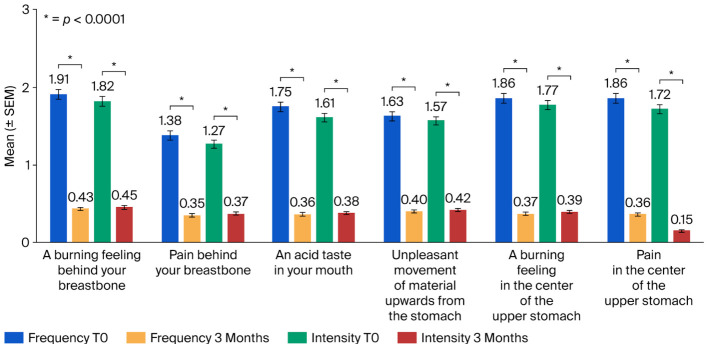
RDQ item-level scores at baseline and after 3 months.

**Figure 3:**
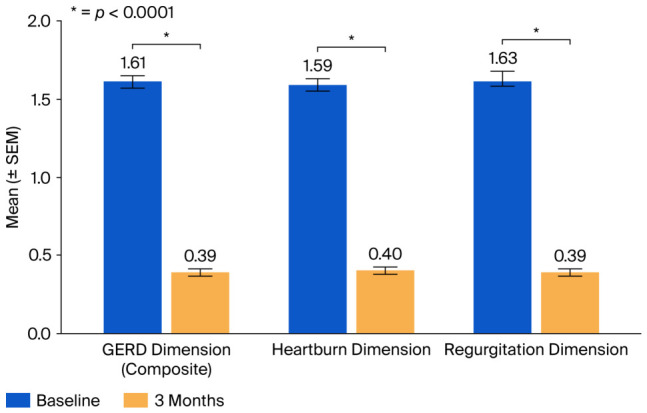
RDQ scores of GERD composite dimension and its constituent heartburn and regurgitation dimension at baseline and after 3 months.

**Figure 4:**
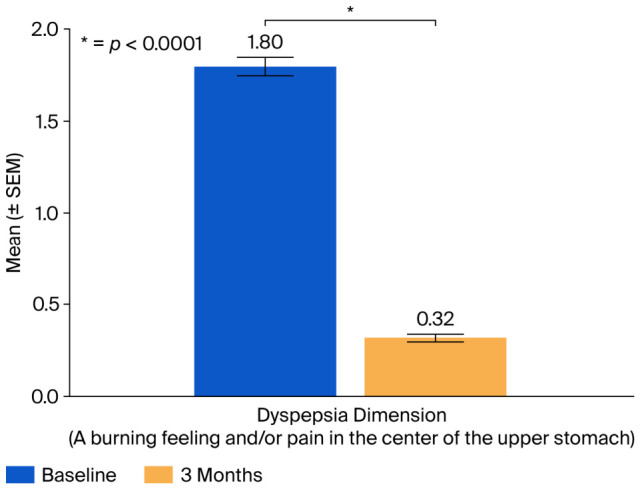
RDQ scores of dyspepsia dimension at baseline and after 3 months.

As shown in Figure 2, all RDQ items showed statistically significant improvement from baseline after 3 months of treatment in both symptom frequency and symptom intensity. Similarly, analyzing the RDQ domains showed statistically significant improvement at 3 months in the GERD composite dimension and in its component dimensions, namely heartburn and regurgitation (Figure 3). A statistically significant improvement from the baseline was also observed in the dyspepsia dimension after 3 months of treatment (Figure 4).

In multivariable linear regression analyses of the change (delta) from baseline to 3 months, female sex was the only variable that remained significantly associated with improvement in the GERD composite, regurgitation, and dyspepsia dimensions, indicating a better therapeutic response in women than in men after adjustment for other factors (Table 2, Table 4 and Table 5). In the heartburn dimension, female sex was also significantly associated with improvement, whereas alcohol consumption was inversely associated with this outcome, with alcohol consumers showing significantly less improvement after adjustment for the other variables (Table 3).

**Table 3:** Multivariate linear regression analysis of changes (⊗) in the heartburn composite dimension of the RDQ from baseline to 3 months (T3–T0).

Variable	Coefficient	95% CI	*p* Value
Age	−0.001	−0.0088 to 0.0055	0.656
Female	−0.2776	−0.4924 to −0.0628	0.011
BMI	−0.0187	−0.045 to 0.0081	0.172
Smoker	−0.1888	−0.4179 to 0.040	0.106
Alcohol use	0.2781	0.032 to 0.5239	0.027

BMI, body mass index; 95% CI, 95% confidence interval.

**Table 4:** Multivariate linear regression analysis of changes (⊗) in the regurgitation composite dimension of the RDQ from baseline to 3 months (T3–T0).

Variable	Coefficient	95% CI	*p* Value
Age	0.0012	−0.0057 to 0.0082	0.723
Female	−0.3576	−0.5679 to −0.1481	0.001
BMI	0.0039	−0.0230 to 0.0294	0.811
Smoker	−0.0445	−0.2679 to 0.1788	0.696
Alcohol use	0.0980	−0.1417 to 0.3377	0.423

BMI, body mass index; 95% CI, 95% confidence interval.

**Table 5:** Multivariate linear regression analysis of changes (⊗) in the dyspepsia dimension of the RDQ from baseline to 3 months (T3–T0).

Variable	Coefficient	95% CI	*p* Value
Age	0.0061	−0.0011 to 0.0134	0.101
Female	−0.2554	−0.4746 to −0.0359	0.023
BMI	−0.0022	−0.0297 to 0.0252	0.872
Smoker	−0.1661	−0.4001 to 0.0671	0.164
Alcohol use	0.2048	−0.046 to 0.4559	0.110

## 3. Discussion

In this large prospective observational study, which included more than 1000 patients with negative UGE findings and negative *H. Pylori* status complaining of upper gastrointestinal symptoms, treatment with the multicomponent formulation Tamacid-Pro^®^ was associated with a significant improvement in all RDQ items. Indeed, both GERD-related symptoms—including the composite GERD dimension and its individual components, heartburn and regurgitation—and gastric symptoms, as assessed by the dyspepsia dimensions, showed statistically significant improvement, thereby supporting the use of a topical, mucosa-targeted therapeutic approach in this patient population.

Patients presenting with reflux symptoms, either alone or in association with epigastric symptoms such as burning or pain, represent a particularly challenging group to evaluate and manage in clinical practice, especially when upper endoscopy is negative and *H. pylori* infection has been excluded [1,2,4]. In such cases, a diagnosis of nonerosive reflux disease (NERD) cannot be presumed solely on clinical grounds, since a more precise pathophysiological characterization would require ambulatory pH-impedance monitoring. Indeed, NERD is characterized by substantial pathophysiological heterogeneity, including abnormal acid exposure, reflux hypersensitivity, and functional heartburn [10]. However, ambulatory pH-impedance monitoring is not routinely available at all centres, being largely confined to secondary and tertiary referral settings. Consequently, these patients are often managed initially with empiric proton-pump inhibitor therapy. Given the substantial pathophysiological heterogeneity of this population and the limited availability of objective diagnostic testing, the limited efficacy of this approach is not unexpected, with up to 40% of patients failing to achieve adequate symptom control [14]. In this context, therapeutic strategies targeting mucosal integrity have gained increasing attention as complementary or alternative options to acid suppression [1,4]. No previous peer-reviewed clinical studies have specifically evaluated the safety or clinical benefits of Tamacid-Pro^®^ in patients with gastroesophageal reflux disease, dyspepsia, or peptic ulcer disease; therefore, the present study provides preliminary clinical evidence on this formulation, while the biological plausibility of its effect is mainly supported by the literature on its individual components and a recent experimental preclinical report suggesting a protective effect of Tamacid Pro^®^ on epithelial integrity in a reflux-related model [15].

The consistent improvement observed across all symptom domains in our cohort is in line with growing evidence suggesting a potential benefit of alginate-based and mucosa-protective formulations in patients with reflux symptoms. Alginates act by forming a physical barrier (“raft”) that reduces postprandial reflux episodes, while additional components may enhance mucosal defence and modulate visceral sensitivity [5]. The multicomponent nature of Tamacid-Pro^®^, combining alginates with plant-derived compounds and a bio-adhesive matrix, could therefore provide synergistic effects through multiple mechanisms, including mechanical protection, anti-inflammatory activity, and modulation of gastrointestinal motility [16,17].

Of particular interest is the significant improvement observed not only in typical GERD symptoms such as heartburn and regurgitation but also in the dyspepsia dimension. This finding further supports the concept of a clinical and pathophysiological overlap between NERD and functional dyspepsia, particularly the epigastric pain syndrome subtype [13]. The observed benefit of Tamacid-Pro^®^ on both reflux-related and dyspeptic symptoms suggests a potential broader therapeutic role in patients with the clinical and endoscopic characteristics of those enrolled in this study, supporting its use either as a first-line approach or as an adjunct to standard therapy.

Another relevant finding of our study is the independent association between female sex and greater symptom improvement across multiple RDQ dimensions. Sex-related differences in symptom perception, visceral sensitivity, and response to therapy have been previously reported in functional gastrointestinal disorders and GERD [9]. Women are known to exhibit increased esophageal sensitivity and a higher prevalence of functional overlap syndromes, which may partly explain the observed differential response [9]. Conversely, alcohol consumption was negatively associated with improvement in the heartburn dimension, in agreement with existing evidence indicating that alcohol may exacerbate reflux symptoms by reducing lower esophageal sphincter pressure and impairing mucosal defence [12].

Our study has several strengths, including the large sample size, the prospective design, and the use of a validated symptom questionnaire (RDQ) to assess treatment response. Moreover, including consecutive patients undergoing endoscopy enhances the real-world applicability of our findings. However, some limitations should be acknowledged. First, the observational design of the study and the absence of a control group limit the ability to infer causal relationships, as the observed improvements may be influenced by confounding factors, placebo effects, or the natural variability of gastroesophageal reflux symptoms. Although symptom assessment was performed using a validated, disease-specific questionnaire and multivariable analyses were adjusted for key demographic and clinical factors, residual confounding cannot be excluded. In particular, detailed information on dietary habits, lifestyle modifications, and concomitant medication use was not systematically collected. The limited number of variables included as covariates in the multivariable models represents a further limitation, as other clinically relevant confounders such as dietary patterns, symptom-trigger foods, body position, concomitant medications, and comorbid psychological conditions were not systematically captured and could have influenced both symptom burden at baseline and the response to treatment. Alsono-standardized lifestyle or dietary intervention protocol was implemented during the study period. General lifestyle and dietary recommendations were provided, when considered appropriate, at the discretion of the treating physician according to routine clinical practice, but these interventions were neither standardized nor systematically recorded. Second, no formal a priori sample size calculation was performed due to the real-world, prospective observational design with consecutive patient enrolment; although the large sample size increases statistical power, the clinical relevance of the findings should be interpreted in light of effect size and consistency across outcomes rather than statistical significance alone. Third, the lack of objective reflux monitoring (e.g., pH-impedance testing) prevents the precise phenotypic characterization of NERD subgroups. Also, the follow-up period was limited to three months, and longer-term data are needed to assess the durability of the therapeutic effect.

Lastly, adherence to treatment was assessed through patient self-reporting, which may be subject to recall bias and could have led to an overestimation of compliance.

In conclusion, our findings suggest that Tamacid-Pro^®^ was associated with improvement in both typical and atypical upper gastrointestinal symptoms in endoscopy-negative patients. While multicomponent, mucosa-protective formulations may represent a promising therapeutic option in patients with GERSs and overlapping functional disorders, further randomized controlled trials are needed to confirm these findings.

## 4. Methods

### 4.1. Patients

Between January 2025 and February 2026, consecutive patients referred to S. Orsola University Hospital in Bologna, Italy, for an upper GI endoscopy (UGE), complaining of upper gastrointestinal symptoms consistent with gastroesophageal reflux and/or dyspepsia, namely heartburn, regurgitation, and epigastric pain or burning, in line with the do-mains assessed by the Reflux Disease Questionnaire (RDQ), were evaluated for this prospective observational study. Treatment with the multicomponent formulation, which was already commercialized, was prescribed at the discretion of the treating physician as part of routine clinical care, and no experimental conditions were imposed beyond standard practice. Inclusion criteria comprised patients aged ≥18 years presenting with upper gastrointestinal symptoms of at least two months’ duration and absence of *H. pylori* infection. Exclusion criteria were (1) age < 18 years; (2) previous upper gastrointestinal surgery; (3) use of proton-pump inhibitors (PPIs) or H_2_ receptor antagonists (H_2_RAs) in the 4 weeks before the endoscopy, a well-established methodological practice in the field to prevent misdiagnosis; patients were either not receiving these medications at the time of enrolment or had been instructed by their treating physician to discontinue them for at least 4 weeks before undergoing upper endoscopy, in order to avoid potential diagnostic interference with endoscopic and histological findings; (4) use of bismuth preparations and/or antimicrobial agents in the 4 weeks before the endoscopy; (5) absence of any visible esophageal mucosal breaks, as defined by the Los Angeles (LA) criteria [21]; (6) patients with severe or unstable general conditions (e.g., cardiovascular, pulmonary, endocrine, renal, hepatic, or hematologic diseases); (7) pregnant or breastfeeding women; and (8) regular use of antacids (defined as use on more than 3 days per week in the 4 weeks preceding enrolment), as this could have confounded symptom assessment and the evaluation of treatment response. As this was a prospective observational study based on consecutive enrolment, a formal a priori sample size calculation was not performed. Patients who declined to participate were not enrolled in the study, and only individuals with complete baseline and follow-up data were included in the final analysis. Patients provided written informed consent for UGE and anonymous use of their clinical data for scientific purposes (i.e., sex, age).

### 4.2. Endoscopy and Biopsies

All enrolled patients underwent a UGE. During the procedure, biopsies were collected for histological examination (2 specimens from the *antrum* and *corpus*, respectively, and 1 from the *incisura*). Biopsies were stained with hematoxylin–eosin and Giemsa stains, and gastritis was scored using the updated Sydney system [21,22].

### 4.3. Urea Breath Tests

Urea breath tests (UBTs) were performed following an overnight fast. Baseline breath samples were collected using 20 mL disposable-straw containers. Subsequently, each patient ingested an aqueous solution containing 1.4 g of citric acid and 100 mg of ^13^C-urea. A second breath sample was obtained 30 min post-ingestion. Samples were analyzed using the new UBT (Helitron V CO_2_—[H_2_] from Nanotech Analysis s.r.l., Turin, Italy [23]). A test was defined as positive if the delta over baseline (DOB) value was ≥5‰ [23]. Patients were defined as negative for *H. pylori* infection if both ^13^C-urea breath test and histological examination yielded negative results.

### 4.4. Treatment and Assessment

Consecutive patients who consented to participate were treated with Tamacid-Pro^®^, which was administered three times daily postprandially (i.e., following breakfast, lunch, and dinner) for a duration of three months. Clinical assessments were performed at baseline and at the 3-month endpoint using the Reflux Disease Questionnaire (RDQ) [24]. Adherence was monitored through patient self-reporting at the follow-up visit, where participants were specifically asked about treatment intake and any missed doses during the study period.

### 4.5. Statistics

Changes in the frequency and intensity of each item included in the RDQ were assessed by comparing baseline values with those obtained at three months. Similar analyses were performed for the GERD composite dimension and for its individual components, namely the heartburn and regurgitation dimensions. In addition, changes in the dyspepsia dimension of the RDQ, defined as a burning sensation and/or pain in the centre of the upper stomach, were evaluated between baseline and three months. For all comparisons, differences over time were analyzed using a paired-samples *t*-test [25]. In addition, the change between baseline and three months (delta) was calculated for the GERD composite dimension, for its individual components (heartburn and regurgitation), and for the dyspepsia dimension. These changes were used as dependent variables in multivariable linear regression analyses including age, sex (female, with male as the reference category), body mass index (modelled as a continuous variable), and alcohol use and smoking status (both dichotomized as yes or no) as independent variables [26]. Proportions, their differences, and 95% confidence intervals (95% CIs) were calculated using the method recommended by Newcombe and Altman [27]. A two-sided *p* < 0.05 was considered statistically significant. Statistical analysis was performed using Stata 16.1 (Stata Corp, 4905 Lakeway Dr, College Station, TX, USA).

## Figures and Tables

**Table 1:** Characteristics of the 1035 patients enrolled in the study.

Female, n (%)	527 (55.3%)
Age, years (SD)	56.4 (14.8)
BMI, kg/m^2^ (SD)	24.9 (3.90)
Active smokers, n (%)	335 (32.4%)
Alcohol consumption, n (%) (at least 1 glass a day)	252 (24.3%)

BMI, body mass index.

**Table 2:** Multivariate linear regression analysis of changes (⊗) in the GERD composite dimension of the RDQ from baseline to 3 months (T3–T0).

Variable	Coefficient	95% CI	*p* Value
Age	−0.0002	−0.0060 to 0.0057	0.952
Female	−0.3176	−0.4945 to −0.1407	<0.0001
BMI	−0.0077	−0.0299 to 0.0144	0.492
Smoker	−0.1166	−0.3053 to 0.0719	0.225
Alcohol use	0.1880	−0.014 to 0.3905	0.069

BMI, body mass index; 95% CI, 95% confidence interval.

## Data Availability

The original contributions presented in this study are included in the article. Further inquiries can be directed to the corresponding author.
